# A Powerful Test of Parent-of-Origin Effects for Quantitative Traits Using Haplotypes

**DOI:** 10.1371/journal.pone.0028909

**Published:** 2011-12-13

**Authors:** Rui Feng, Yinghua Wu, Gun Ho Jang, Jose M. Ordovas, Donna Arnett

**Affiliations:** 1 Department of Biostatistics and Epidemiology, University of Pennsylvania, Philadelphia, Pennsylvania, United States of America; 2 Nutrition and Genomics Laboratory, JM-USDA Human Nutrition Research Center on Aging, Tufts University, Boston, Massachusetts, United States of America; 3 Department of Cardiovascular Epidemiology and Population Genetics, Centro Nacional de Investigaciones Cardiovasculares, Madrid, Spain; 4 Department of Epidemiology, The University of Alabama at Birmingham, Birmingham, Alabama, United States of America; Aarhus University, Denmark

## Abstract

Imprinting is an epigenetic phenomenon where the same alleles have unequal transcriptions and thus contribute differently to a trait depending on their parent of origin. This mechanism has been found to affect a variety of human disorders. Although various methods for testing parent-of-origin effects have been proposed in linkage analysis settings, only a few are available for association analysis and they are usually restricted to small families and particular study designs. In this study, we develop a powerful maximum likelihood test to evaluate the parent-of-origin effects of SNPs on quantitative phenotypes in general family studies. Our method incorporates haplotype distribution to take advantage of inter-marker LD information in genome-wide association studies (GWAS). Our method also accommodates missing genotypes that often occur in genetic studies. Our simulation studies with various minor allele frequencies, LD structures, family sizes, and missing schemes have uniformly shown that using the new method significantly improves the power of detecting imprinted genes compared with the method using the SNP at the testing locus only. Our simulations suggest that the most efficient strategy to investigate parent-of-origin effects is to recruit one parent and as many offspring as possible under practical constraints. As a demonstration, we applied our method to a dataset from the Genetics of Lipid Lowering Drugs and Diet Network (GOLDN) to test the parent-of-origin effects of the SNPs within the PPARGC1A, MTP and FABP2 genes on diabetes-related phenotypes, and found that several SNPs in the *MTP* gene show parent-of-origin effects on insulin and glucose levels.

## Introduction

Family data have been extensively collected and analyzed in the early stage of gene mapping or linkage mapping studies and some family-based studies have been updated with new genotype data to meet recent interest in association mapping. Extra valuable LD information has been obtained in addition to the traditional linkage analysis. Family-based studies are exempt from population stratification and can provide valuable prior knowledge for gene–gene and gene–environment interactions [1]. Unique to family data is that one can study parent-of-origin effect, and in this work we introduce a new powerful method using haplotypes to test the parent-of-origin effects of SNPs- on quantitative traits.

Imprinting is a crucial epigenetic phenomenon where the same alleles have unequal transcriptions and thus different contributions to a trait. The presence and magnitude of the effect of an allele copy depend on whether it is inherited from the father or the mother and thus the effect is often called parent-of-origin effect. The parent-of-origin effects of imprinted genes have been observed in various human diseases including cancer [2], type I diabetes [3], [4], and bipolar disorder [5], [6]. Although many associations between genetic variants and human traits have been discovered through genome-wide associations, the impact of parental origin has largely been ignored. In Kong et al. [7], at a locus at 11p15 associated with type 2 diabetes, the same allele can confer risk if paternally inherited and decrease risk if maternally transmitted, providing solid evidence for the parent-of-origin effect with sequence technique.

The key to investigate the parent-of-origin effect of a gene on a trait is to distinguish maternally and paternally transmitted alleles; therefore, family-based studies are necessary. Statistical methods were developed to test the parent-of-origin effects on human diseases more than a decade ago. Most of these methods are extensions of linkage analysis methods intended for sparse microsatellite markers. For binary traits, Strauch et al. [8] introduced additional penetrance parameters to the classic parametric linkage model to account for parent-of-origin effects, and established the likelihood ratio test (LRT) under the hypotheses of equal parental contributions vs. unequal contributions. However, without prior information, specification of a disease model may be heuristic especially for genome-wide scans. As maximizing the likelihoods over all possible disease allele frequencies and penetrances could result in irregular distribution of the LRT, the statistical asymptotic theory may be inapplicable [9]. For quantitative traits, variance component (VC) methods have been expanded to separate the genetic variance into two components, one due to maternal alleles and the other due to paternal alleles. The specification of the variance structure requires the estimate of the probability of parent-of-origin-specific allele-sharing identical by descent (IBD) [10], [11], [12]. For sibling pairs, the Haseman-Elston regression method [10] has been modified to regress on separate parent-specific IBDs. For trios, Whittaker et al. [13] used a linear model that can accommodate maternal effects, offspring genotypic effect, and parent-of-origin effect. Extensive evaluation and comparisons have been conducted on both regression-based and VC methods in linkage analysis and the VC methods are often favored for their higher power than regression-based procedure, especially in extended pedigrees [14], [15], [16], [17], [18], [19], [20]. Originally proposed for linkage analysis, these methods often have low power due to the sparse coverage of microsatellite markers and available family size. Most of the methods are limited to siblings, relative pairs, or case-parent triads [10], [21], [22], [23], [24]. Only a few, essentially VC and variants of VC, can be applied on extended pedigrees, which contain more inheritance information than small families [8], [11], [25]. The VC method [11] using the extended pedigrees has been compared with the parent-of-origin method for sibship data [10], [12], with the former used the family information more efficiently and thus has higher power. However, the calculation of parent-specific IBD is generally computationally intensive for extended pedigrees, which have also prevented these methods from wider applications.

Using haplotype and phase information can increase the accuracy of IBD estimation compared to using only genotype information [26], but still the full IBD information cannot be recovered completely. Haplotype frequency estimates can be improved using pedigrees over using unrelated individuals only and such improvement can often affect the disease association findings [27], [28], [29], [30], [31]. Along the TDT line in triods, Cordell *et al*. [32] contrasted the haplotypes of cases and pseudo-controls to detect parent-of-origin effects. However, this method used only those data where the parental haplotypes can be unambiguously deduced without recombinants or all families in which the haplotypes are “inferable” may discard many families and result in loss of power.

Statistical methods for association can be more powerful than linkage because of the use of specific alleles rather than IBD in linkage [33] and abundant methods have been proposed for association studies, but only a few can test parent-of-origin effects in an association study setting. Weinberg [24] proposed a log-linear model based on stratification on both the parental mating type and the inherited number of alleles in case–parent trios. For quantitative traits, Whittaker et al. [13] adopted a three-way ANOVA model that classifies the parent–triads according to their genotype combinations and includes an additional term with transmitted paternal alleles to test parent-of-origin effects. These methods are all single-locus models and do not make use of the valuable intra-marker information contained in GWAS; they are also based on small pedigrees and limited to certain designs. To the best of our knowledge, no method is available to test parent-of-origin effects in the most informative extended pedigrees.

In this work, we have developed a maximum likelihood method to test parent-of-origin effects on a quantitative trait using all phenotype and genotype information from all relatives in a pedigree. In this approach, genotype data at adjacent markers and the intra-marker LD information are used to infer the parent of origin of nonfounders' alleles and thus the power for testing parent-of-origin effects is expected to improve over the method using only the genotype at the testing locus. In essence, the single-locus method is just a special case of the haplotype-based method with the block length of one, which is the least informative. The methods are illustrated for several nuclear family sizes, and different haplotype structures in the simulation section.

In family studies, missing genotypes and phenotypes of founders are common due to the late-onset of the disease, the geographical limits, failed informed consent, single-parent families, etc. Even for small families where all the data can be easily collected, missing genotypes can still occur in a more random pattern due to the genotyping techniques [34]. We extended our method to accommodate missing data. If one person has missing genotypes, his/her relatives' genotypes are used to improve the estimate of haplotype frequency and the inference of haplotype origins.

For large pedigrees, we developed a revised Elston-Stewart algorithm, which starts with the bottom generation and peels the likelihood of pedigrees into sequential conditional probabilities to ease the computation. Pertinent to the model incorporating parent-of-origin effect, both transmission and penetrance probability are determined by the haplotypes and their origins.

Our methods are developed and evaluated for quantitative traits, but they can be easily extended to binary and ordinal traits in the framework of generalized linear models.

## Methods

### 1) Notation

At a testing SNP locus, we denote a minor allele by *a* with its frequency 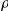 and a major allele by *A*. We use two adjacent letters, AA, Aa, and aa for the possible genotypes at this locus and comma-separated pairs, (A, A), (A,a), (a,A), and (a,a), for the sourced genotypes with the first letters indicating the paternal copies and the second indicating the maternal copies. For convenience, we also use binary digits to code alleles, with 0 for *A* and 1 for *a*, and thus (0,0), (0,1), (1,0), and (1,1) for the 4 coded sourced genotypes, respectively. We assume that a haplotype block containing the testing locus has a length *L* and a total of *t* possible haplotypes *h_1_*,…, *h_t_* with population frequencies 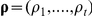. We denote the pair of the unsourced haplotypes (so-called “diplotype”) of an individual as *h/h′*, and the sourced haplotypes as (*h, h′*). In every family, *n_i_* is the number of nonfounders and *f_i_* is the number of founders, *G_0_* is the genotype set of all nonfounders, and *G_f_* is the genotype set of all founders.

### 2) Model for Test of Parent-of-Origin Effects

We consider a quantitative trait 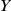. Let 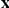 be a vector of covariates (including intercept). Given each individual's coded sourced genotype (*g_p_, g_m_*), the traits of all the nonfounders follow
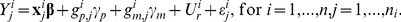
(1)where *n* is the total number of pedigrees, *i* is the family index, *j* is the individual nonfounder index within a family, 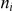 is the number of nonfounders in the 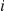 ^th^ family, 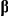 is a vector of parameters reflecting the covariate effects on the trait, 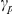 and 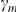 are the genetic effects corresponding to paternal and maternal alleles, respectively, 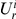 is a random effect following the multivariate normal distribution with the 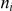
×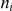 scaled variance–covariance kinship matrix 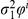 within the *i^th^* family (specifically, the element of the *j* ^th^ row and *l* ^th^ column of 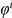, 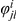 where 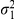 is the polygenetic variance and 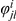 is the kinship coefficient between two nonfounders *j* and *l* in the *i^th^* family that is completely determined by the their relationship without knowing any genetic marker), and the random error 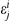 is assumed to follow a normal distribution with mean 0 and variance 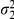. The null hypothesis of interest is that the effects from paternal and maternal alleles are equal, i.e., 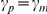. Model (1) can be written as an equivalent model
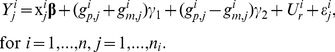
(2)Where 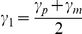 can be regarded as the main genetic effect and 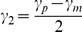 represents the parent-of-origin effect. Under the null hypothesis of no parent-of-origin effect, 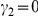, the model becomes 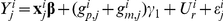, which is a general additive model to test association. We will use model (2) so that the hypothesis testing becomes straightforward. Analogous to the interpretation of 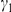, the sign of 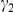 implies the direction of parent-of-origin effect: a positive 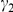 means that the maternal allele *a* is associated with larger phenotypic value and a negative 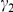 means that the paternal *a* is more associated with larger phenotypic value. In addition, the relative magnitude of 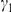 and 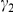 reveals how balanced the maternal and paternal effects are. In the extreme cases, 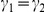, (i.e., 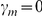) implies complete silence of maternal allele *a* that is functional otherwise, and on the other hand, 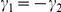, or 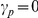, implies complete silence of paternal allele *a*.

Our proposed model is similar to the models proposed by Weinberg et al. (1999) for case-parent-triads design, which also formed the contrast of transmitted paternal and maternal alleles, but in cases only. Our model, however, can be applied to general families. In addition, the genotype combination that leads to ambiguous inference of parent of origin of child's alleles was treated as a separate class in Weinberg et al. (1999), while our model considers all the possibilities including ambiguous states even with missing data, and thus, uses the maximum information in the likelihood calculation.

### 3) Likelihood

Given all individuals' genotypes, the likelihood of family *i* is a function of 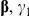, 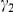, 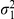, 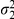 and haplotype frequencies and is given by
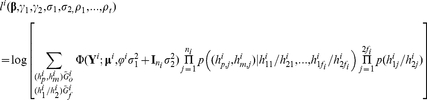
(3)where *j* is the individual index within the family, 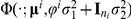 is the multivariate-normal probability density function with mean vector 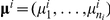, 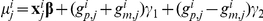, for 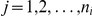 and variance 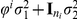, 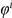 is the kinship matrix, 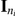 is the 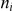
×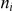 identity matrix, 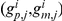 at a testing locus is a subset of the sourced haplotypes 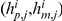 and thus can be uniquely determined. Under the assumption of Hardy–Weinberg Equilibrium (HWE) for the haplotypes, we have 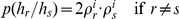 
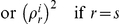
.
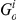
and 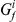 refer to the set of offspring and founders' genotypes at all loci within the haplotype block. Please note that we aim to test the parent-of-origin effect at a testing locus but not the parent-of-origin effect of any haplotype. Instead, we borrow the haplotype information to help identify the parent of origin of the allele at the testing locus. To better understand how using haplotypes improves the power, we can consider a simple example of a trio at two loci, with possible genotypes A/a and B/b. If mother, father, and child's genotypes are AaBB, AabB, and AaBb, respectively, we would be unable to know the parental source of alleles A and a of the child. But if we know that there are only three possible haplotypes AB, Ab, or aB, i.e., *π*(ab) = 0 in the population, we can then infer the sourced haplotypes of the child as (Ab, aB) and thus the sources of A and a are determined.

The likelihood for all the data would be 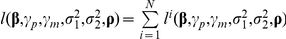 and maximizing it yields the MLE of the parameter of interests, including covariate and genetic effects, phenotypic variance, and minor allele frequency or haplotype frequency.

Since direct calculation of the likelihood function is computationally intensive, a revised Elston-Stewart Algorithm [27] can be used instead, which processes nuclear families from the latest generation of the pedigree and then traces back to earlier generations (see Appendix S2).

### 4) The EM Algorithm for Estimating Haplotype Frequencies, Variances, and Genetic Effects

When the number of possible haplotypes *t* is large, maximizing the likelihood (4) over a large number of parameters may present daunting convergence problems and require too much effort. To improve the computational efficiency, we develop an iterative EM algorithm. Given a set of initial values of the parameters, the algorithm estimates the parameters by repeating the following E-step and M-step until convergence:

- At the *k* ^th^ iteration, calculate the conditional probabilities of all haplotypes by counting all possible haplotype pairs of founders that are compatible with observed family members' genotypes and update the estimates of haplotype frequencies by the following equation, which aggregates the probabilities *p*(*h/h*′),
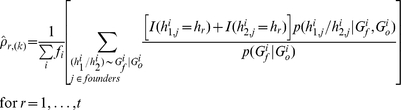
where *f_i_* is the number of founders in family *i*, the denominator can be decomposed as 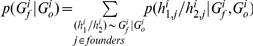, and under HWE, we have 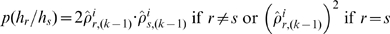.
- At the k^th^ iteration given 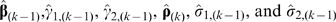, update the 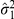 and 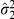 by solving 
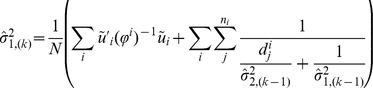
 and 
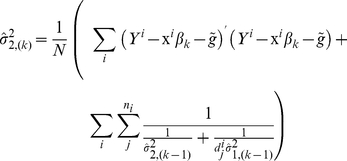
where 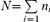, 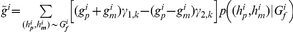, 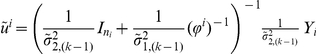, and 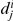 is the *j^th^* diagonal element of the diagonal matrix from the singular value decomposition of the kinship matrix 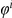 of family *i* (Derivations in Appendix S1).
- Maximize the likelihood function (4) with respect to 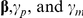
while fixing 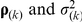.

Step (1) is the E-step and steps (2) and (3) are the M-step. This EM algorithm is relatively robust for departures from HWE and is easy to implement [35]. A sensible ensemble of the initial parameters can be the effect estimates from the association model using complete data and frequency estimates based on founders' genotypes.

### 5) Likelihood Ratio Test (LRT)

Our test statistic is a likelihood ratio statistic, i.e., twice of the difference between the maximum log-likelihood under the null and alternative hypotheses. The statistic follows the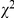 distribution with one degree of freedom and thus 100(1-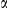) percentile of
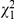
is the critical value for rejecting the null at the significance level of 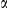. Both the Elston-Stewart and EM algorithms are similarly implemented under the null hypothesis as under the alternative hypothesis except that the parameter γ_2_ does not appear under the null.

### 6) Missing Genotypes

In family studies, the information of founders or older generations is more likely to be missing. For founders, it is impossible to tell the parent of origin of their alleles and thus their genotype and phenotypes are not particularly useful in estimating the parent-of-origin effect. But their genotypes are useful for referring the parents of origin of the alleles of their offspring. When a founder has missing genotypes, we may lose some accuracy in inferring the parents of origin of the alleles of their offspring. In this case, the parent of origin of the offspring's alleles may be inferred from one parent (the founder's spouse)'s genotypes. Sometimes the missing genotype information can be completely recovered by using the offspring and spouse's information. Such recovery can be more efficient when more neighboring loci information in the population can be borrowed. Generally, the more relatives genotyped, the more accurately we can infer haplotype phases and allele origin information as the genotypes of other pedigree members can give clues to determine missing genotypes.

If not all missing genotypes can be recovered, the likelihood function in Eq. (5) remains the same except that the set of haplotypes compatible with observed genotypes may increase to account for more possibilities due to the missing genotypes. The size increase of the compatible set applies to both single-locus and haplotype-based methods, but when inter-marker information is taken into account in the haplotype-based method, the increase might be much smaller compared with using only genotypes at the single testing locus. The likelihood with missing data is given by
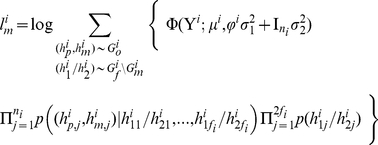
where 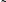 is a notation for compatibility meaning that the haplotypes on the left are compatible with the founder and offspring genotypes and Mendelian inheritance, and “\G_m_” means excluding those missing genotypes. For example, 3 possible haplotype pairs “100/100,” “100/110,” and “110/110” are consistent with observed genotypes “2?0” of a founder, denoted by “100/100, 100/110, and 110/110∼2?0,” where “?” denotes the missing allele.

*A note about the likelihood using only testing locus*

This would be a special case of haplotype-based method and the log-likelihood function for the *i^th^* family can be simplified as,
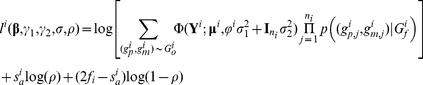
(4)where 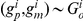 means the possible sourced alleles of all nonfounders in the *i^th^* family compatible with their observed genotypes 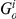, 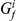
is the set of founders' genotypes, and 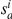 is the total numbers of allele *a* carried by all the founders in the *i* ^th^ family. The conditional probability 
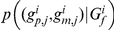
 is essentially the inheritance probability. This likelihood is only concerned about the genotypes at the testing locus and thus the effective set
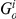
and 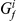 is the genotype set at the testing locus.

### 7) Simulations

We conducted simulation studies to evaluate the performance of our method using only alleles at the testing locus for various family sizes, heritability, and minor allele frequencies, which serves as the baseline for comparison with our haplotype-based methods. We then investigated influence of the length of the haplotype block and the LD between the testing locus and adjacent loci on the power of the haplotype-based method. We also inspected our approaches under different missing mechanisms and compared efficiency across study designs.

In each simulation study, we simulated 200 nuclear families with *m* (*m = 1, …,4*) offspring in each family. For the model using the genotypes at the testing locus, the genotype of founders was generated based on a SNP locus with a minor causal allele and then the genotypes of their offspring were generated assuming random mating and Mendelian inheritance. The quantitative phenotypes of all the offspring were generated according to the true model (1). Defining the additive genetic inheritance *h* as the proportion of phenotypic variance explained by the causal SNP, we let the maternal allele and the paternal allele at the causal SNP locus explain *80h%* ( = *ρ*(1-*ρ*)*γ_m_^2^*) and *20h%* ( = *ρ*(1-*ρ*)*γ_p_^2^*) of the phenotypic variation, respectively, and the residual genetic variation equal 10%. The parent-of-origin effect (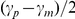) was reflected in the unbalanced heritability due to the paternal and maternal alleles, i.e., the difference in variances in Y explained by the paternal and maternal alleles. Then we used our likelihood ratio tests to test parent-of-origin effect. We let the frequency of the causal allele be 0.1, 0.3, and 0.5 and let the additive inheritance *h* change from 0.025, 0.05, 0.1, to 0.2, respectively. In the simulation of parent-trios (*m = 1*), the polygenetic effect cannot be estimated with only one offspring in each family and thus model (2) degenerated to a fixed effect model without 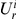.

To evaluate the haplotype-based method, we generated the genotypes of 5 SNPs based on the haplotype structure in gene GPX1, a well-known gene for encoding a member of glutathione peroxidase, an important antioxidant enzyme in humans. For the 5 loci within the GPX1, there are 7 common haplotypes with their frequencies and pair-wise LD shown in Table S2 [36]. Assuming HWE, the founders' haplotypes were generated based on the GPX1 haplotype frequencies. In each family, the offspring's haplotypes were inherited following the Mendelian law and assuming no recombination within the block. To allow some variation in the LD between the testing SNP and neighboring markers, we chose each of the 5 SNPs in turn to be the causal SNP. The phenotype was generated similarly as before except that we fixed the heritability to be 10% and let the maternal and paternal alleles affect the phenotype in the same direction and explain 8% and 2% of the total phenotypic variance, respectively. We tested the parent-of-origin effect at the true causal locus using only testing locus and the haplotypes within the different blocks.

To compare with existing methods, we also tested each locus for parent-of-origin using the VC method [11], which has been claimed to be the most powerful for pedigree data so far. To maximize the information utilized in VC, parent-specific IBD distribution was estimated based on the whole haplotype block and was used in the variance structure of paternal and maternal components respectively.

To assess the information loss at the presence of missing genotypes, we further investigated the performance of our test statistics under two common missing scenarios. First, we considered the situation where one parent in each family was not genotyped and thus his/her genotypes over the genome were completely missing. So, the genotypes of the other parent and their offspring can be useful to infer the phases and parent of origin of their haplotypes. Second, we mimicked the sparse genotype missing due to genotype calling algorithms or errors and let the missing rate be a uniform 10% across all loci. Depending on the LD between the loci with missing genotypes and neighboring locus, the missing alleles and their origin can be partially or completely inferred.

Last, to get a clue about the most efficient strategy for genotyping in the context of testing parent-of-origin effect, we examined the power for different family structures while keeping the same total number of 1200 individuals. Specifically, we checked families with both parents and 1 to 4 offspring, families with a single parent and 1 to 5 offspring, assuming all parents have phenotypes and 10% of offspring have missing phenotypes to mimic the situation in real studies.

For each fixed set of parameters, each simulation experiment was repeated 5000 times for the type I error assessment (*h* = 0) and 1000 times for the power (*h*>0).

### 8) Real Data Analysis

To demonstrate the capabilities of our method in studies involving large pedigrees and the advantage of using the haplotypes, we tested the parent-of-origin effects on diabetes-related phenotypes, specifically the HOMA, insulin, and glucose levels, using the Genetics of Lipid Lowering Drugs and Diet Network (GOLDN) study data. The GOLDN study recruited 3-generation families from two NHLBI Family Heart Study (FHS) field centers in Minneapolis, Minnesota and Salt Lake City, Utah that included 661 families with the highest risk scores and early onset of CHD and 592 randomly sampled families. In addition, GOLDN also recruited offspring of the original FHS probands' siblings and relatives who were not included in the original FHS sampling [37]. Most families have 2–3 generations with 5–20 individuals and 60% of those who were eligible to participate completed the study protocol, which was approved by the Institutional Review Boards at the University of Minnesota, the University of Utah, and Tufts University.

The initial aim of GOLDN was to identify the common genetic and environmental factors for the plasma triglyceride (TG) response to a TG-raising diet and a lipid-lowering drug—fenofibrate. Exclusion criteria included recent history of heart, liver, kidney, pancreas, and gall bladder diseases; malabsorption of nutrients; current use of insulin or warfarin; high fasting TGs; high serum concentrations of aspartate aminotransferase; high serum concentrations of alanine transaminase, or low glomerular filtration rate; and pregnant or nursing women. See Lai et al., 2007 for details. Written informed consent was obtained from each participant.

Serial clinical measurements, including post-prandial lipemia (PPL), fasting TG, NMR measures of particle size, RBC fatty acids, insulin, glucose, and adiponectin were collected during the visits before and after exposure to fenofibrate for 872 individuals in 176 Caucasian pedigrees. Weight, BMI, demographic and lifestyle information, medical history, current prescription, and medication use, were measured or collected for the participants.

Tag SNPs within candidate regions for various TG-related phenotypes were genotyped with the Applied Biosystems TaqMan SNP genotyping system. A total of 109 SNPs in 23 candidate genes on 14 chromosomes were genotyped. Haplotype blocks within each gene were identified using Haploview [38]. The assumption of Hardy–Weinberg Equilibrium was checked for all SNPs to be tested.

As an illustration of utility of our method, we picked chromosome 4, which harbors 3 candidate genes, to investigate the parent-of-origin effects of these SNPs on the Homeostatic Model Assessment (HOMA, an insulin resistance index), insulin, and glucose levels. We examined the distributions of the phenotypes across different factors and their association with continuous variables. We first fitted linear models for the phenotypes with all possible demographic, clinical, and environmental predictors known in the literature, including age, gender, BMI, center, alcohol drinking, smoking, physical activities, and computer-TV hours. Significant predictors were then retained in the model for genetic analysis.

To reduce the computation time, we used MERLIN [39], which used an efficient sparse gene flow trees algorithm to store and evaluate the inheritance vectors. Merlin found all possible combinations of phased haplotypes compatible with the pedigree information, which we collapsed into unique sets of haplotypes within each gene and then used in our haplotype-based model (2). The probability for each combination was calculated based on the likelihood function. The haplotype frequencies and effects for covariates, genotype, and parent-of-origin were estimated based on MLE. To check the power gain achieved by using the whole haplotype block, we also tested parent-of-origin effects using the single testing locus only after adjusting for confounders and significant predictors in the model.

## Results

### 1) Type I Error Rates

The type I error rates of the methods using the testing locus only and haplotypes at the significance level of 0.05 for different family sizes are summarized for results at locus 5 in Table 1. The results were similar for different loci, haplotype block size, and family sizes, and the type I error rates were consistent with the nominal level for various scenarios. Because multiple loci are often tested simultaneously and the significance level is often adjusted for multiple testing, we also checked the type I errors at significance levels of 0.01 (Table S1) and 0.001 (data not shown). All the empirical type I errors were in accordance with the nominal rates, suggesting that both the single-locus and the haplotype-based methods are valid.

**Table 1:** Type I errors of test of parent-of-origin effect in nuclear families with different family sizes and different missing mechanisms, using different haplotyple block length, at α = 0.05.

Family size	Missing Mechanism	Using haplotypes with different block length L			
		L = 1	L = 2	L = 3	L = 4/5
1 offspring	(i) no missing	0.050	0.047	0.054	0.050
	(ii) 10% random missing	0.055	–	–	0.051
	(iii) one parent missing	0.050	–	–	0.052
2 offspring	(i) no missing	0.048	0.057	0.055	0.049
	(ii) 10% random missing	0.055	–	–	0.049
	(iii) one parent missing	0.052	–	–	0.050
3 offspring	(i) no missing	0.049	0.053	0.050	0.052
	(ii) 10% random missing	0.052	–	–	0.052
	(iii) one parent missing	0.057	–	–	0.052
4 offspring	(i) no missing	0.049	0.053	0.050	0.049
	(ii) 10% random missing	0.055	–	–	0.047
	(iii) one parent missing	0.046	–	–	0.048

### 2) Power

**Figure 1:**
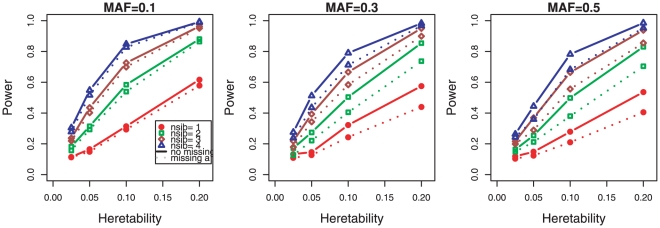
Power of detecting parent-of-origin effect in nuclear families using the testing locus only. Three causal minor allele frequencies (MAFs) are considered and are shown is three separate pannels. Four family sizes are considered and the numbers of siblings 1–4 are indicated by different colors red, green, brown, and blue. Solid lines are for no missing (complete) parental genotypes, and dotted lines are for one parent's genotype missing.

The power of our method using the testing locus only is shown in Figure 1 for different heritability, family sizes, and missing mechanisms. Clearly, the power to detect a significant parent-of-origin effect increased with the increase of heritability at the testing locus and the family size. Since heritability is determined by both MAF and effect size and increased as either one does, given the same heritability, there is a trade-off balance between the MAF and the effect size. Inferring parent of origin of offspring's alleles can be affected by the MAF at the testing locus and thus we also inspected the power at different MAF shown at the three panels in Figure 1, which showed that the power was slightly lower at larger MAF but it was not nearly as sensitive as to heritability.

**Figure 2:**
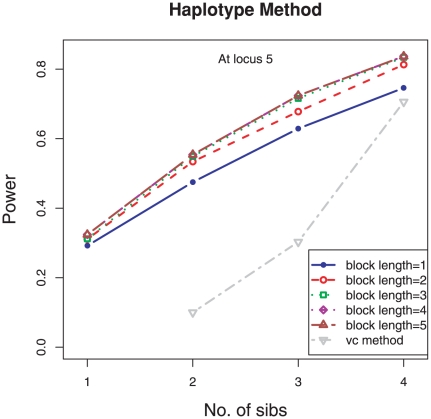
Power of detecting parent-of-origin effect using different haplotype block lengths for different family sizes. The length of haplotype blocks 1–5 are indicated by different colors blue, red, green, purple, and brown. They are compared with the power using the variance components (vc) method, shown by grey color.

Figure 2 compares the power of detecting the parent-of-origin effect using various haplotype lengths, from 1 to 5. We only presented the results for the last SNP as the causal SNP and the results for other loci were similar (Figure S1, blue lines). The benefit in using longer haplotype length is that with longer haplotype blocks, there are more haplotypes giving the same genotypes and thus it is easier to infer the parent of origins of those offspring's alleles, which would be ambiguous using the testing locus only or shorter blocks. Figure 2 demonstrates that the method using the haplotypes improved the power over the method using the testing locus only and longer haplotype blocks led to more power gain. Figure 2 also shows that it is not necessary to use a haplotype block that is too long because when the number of compatible haplotypes stops increasing the power will remain the same.

Compared with the variance component method (Figure 2, grey line), our method shows great improvement of power, which is expected as our model for association analysis uses specific alleles and variance component method uses the IBD shared between individuals that is inferred from individual allelic information but cannot recover all information contained in all alleles.

In our simulation study, the power increase was 11.0%, 16.6%, 15.1%, and 12.2% for the family size ranging from 3 to 6, respectively. The pattern was consistent across all loci (Figure S1), and we can interpret it as the following. When the family size is small, it is not easy to infer parents of origin, even with the help of haplotypes; and when the family size is large, it is already easy to infer parents of origin from siblings even just considering single locus. Figure 2 suggests that the haplotype-based method is most advantageous for families having three or four children. Nonetheless, the method using haplotypes gave at least 10% power gain over the method using the testing locus only.

**Figure 3:**
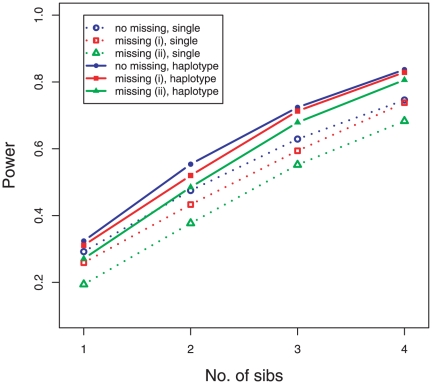
Power of detecting parent-of-origin effect with and without missing genotypes. Three scenarios are considered: no missing, (i) missing one parent's genotype in each family, (ii) for 10% random genotype missing, shown in blue, red, and green, respectively. Results connected by solid lines are obtained by the method using haplotypes, and the results connected by dotted lines are obtained by the method using the testing locus only.

Since the information contributed by haplotypes depends on the LD structure among the SNPs, there is no doubt that the LD structure could influence the power gain when using our haplotype-based method. We can look at two extreme circumstances to get an intuitive understanding of the consequence of LD on the tests. When all neighbor marker loci are in complete LD with the testing locus (R^2^ = 1), the method using the haplotypes within the block is equivalent to the one using any single SNP locus and doesn't contribute additional information for inferring the parent of origin of the alleles at the testing locus even though two possible haplotypes can be easily inferred in this case. The additional marker loci can be useful to infer the genotypes at the testing locus when genotypes are missing at random, but such a contribution to the test of parent-of-origin effect is minor. On the other hand, when all other loci are in linkage equilibrium with the testing locus (R^2^ = 0), they are not useful for inferring haplotypes and thus cannot help inferring parent of origin either. So with other conditions being identical such as heritability, MAF, haplotype block length, and family structure, the largest power gain using haplotypes vs. the single locus genotypes should be when the causal locus's LDs with others lie in the middle range between 0–1. In our simulation, the largest power gain was at locus 5 (Figure 3 and Figure S1) whose average R^2^ with other loci is 0.16 (out of 0.3, 0.16, 0.01, and 0.16). It is noticeable that for a family size of 4, the power gain at locus 4 was larger than at locus 2, both of which had similar MAFs (0.149 and 0.151) but the average R^2^ at locus 4 (0.271) was slightly larger than that at locus 2 (0.198). In this case, a slightly higher LD between the testing locus and adjacent markers seems to have helped us recover the parent of origin information of the alleles at the testing locus. However, because the interplay of pair-wise LDs, marker MAF, haplotype frequencies, and block length is complicated, it's difficult to describe the exact relation between LDs and power. In addition to the GPX1 gene, we also performed simulation studies on gene IGF2 and gene ASAH1. IGF2 is a gene well known for maternally imprinting that contains 6 SNPs in low LD of each other (R^2^ from 0.008 to 0.806). ASAH1 is a long gene that has been found associated with lung cancer and Farber's Disease [40], and there are 14 tagged SNPs in high LD and 9 of them are in complete LD (majority of pair-wise R^2^>0.71). We observed a similar scale of power gain using the haplotype-based method over the single-locus method in both simulations.

Figure 3 shows the power under two different missing scenarios for different family sizes, (i) for missing one parent in each family, (ii) for 10% random missing. While loss of power was inevitable under both scenarios, the impact due to missing parents was more substantial for the method using the testing locus only, as shown in Fig. 1. The power loss was hard to recover even with the haplotype-based method. But it can be recovered better using the design with larger families. We also noticed that the type of power loss was more dependent on the MAF (Figure S1), which was smallest when the MAF at the testing locus is close to 0.15, and largest when the MAF was close to 0.5. However, when only occasional random genotypes are missing, the power of the method using the haplotypes could reach almost the same level as with complete data, suggesting that random missing caused by genotype errors or different platforms is more recoverable from neighboring SNPs. The same reasoning underlies genotype imputation methods.

Summarizing Figures 1,2,3, we found that the power to detect parent-of-origin effect increased almost linearly with increasing family size, and using the haplotype-based method could significantly improve the power under all the situations considered.

### 3) Optimal Family Size for Testing Parent-of-Origin Effects

We checked the power using different numbers of siblings (1–5) and parents (1 or 2) in a family while keeping the total number of individuals the same at 1200. Table 2 tabulates the results for various combinations. Clearly, genotyping more family members within families could lead to larger power given the same total number of individuals and, interestingly, the power gain was most efficient when we genotyped one more offspring in sacrifice of a parent. This conclusion is the same as in the association analysis (Chen et al., 2007). So, the most cost-effective strategy is to genotype one parent and as many offspring (here maximum 5) per family as possible. This choice of family members provided better information about the phases for the haplotypes segregating in the family, and allowed our haplotype-based method to take advantage of adjacent SNP data to fill in the missing genotypes for the missing parent. This was different from the most-effect genotyping strategy for association analysis, which is to examine one offspring and one parent (Chen et al., 2007). Without much risk, one can generalize the conclusion to extended families that we can gain the most power with large families. Most of the family structures in the GOLDN data fall into this category, providing an ideal case for test of parent-of-origin effects.

**Table 2:** Power of detecting parent-of-origin effect using 1200 individuals with different family structures and with/without missing phenotypes.

			Locus 1		Locus 5	
	Parent	No. of sibs (no. of families)	Using testing locus only	Using haplotypes	Using testing locus only	Using haplotypes
Complete Phenotype	both	1 (400)	490	560	489	543
		2 (300)	667	730	636	718
		3 (240)	771	826	710	800
		4 (200)	788	848	772	841
	single	1 (600)	603	675	545	589
		2 (400)	748	820	664	740
		3 (300)	823	865	738	831
		4 (240)	846	888	780	857
		5 (200)	856	911	788	884
10% Missing Phenotype	both	1 (400)	483	528	439	499
		2 (300)	639	693	580	644
		3 (240)	721	770	652	742
		4 (200)	756	803	709	785
	single	1 (600)	568	662	502	560
		2 (400)	701	783	625	725
		3 (300)	775	828	706	775
		4 (240)	795	837	713	824
		5 (200)	818	879	754	854

Also from the bottom half of Table 2, the power loss due to 10% of missing phenotypes for the offspring using the haplotype-based method was generally smaller than using the single-locus method. This is because the offspring's genotypes can still be useful to infer the parents of origin of other siblings' testing alleles even though they do not contribute their phenotype probabilities to the likelihood.

### 4) Real Data Analysis Results

The 45 individuals that are not consanguineous with any pedigree members were removed from the analysis. The remaining data included 1709 individuals within 161 pedigrees. The pedigree sizes range from 3 to 38. Three pedigrees have more than 32 individuals and each was separated into 2 smaller families; five pedigrees have a modest number of members ranging from 20 to 30 and they were trimmed into smaller ones by removing one or two individuals without informative phenotypes or the youngest members. These preparations were theoretically not necessary and would probably affect the power slightly. They were done due to its memory constraint as we used MERLIN to infer possible haplotypes for extended families, taking advantage of the available and well-tested software. The inferred haplotypes would then be fed into our haplotype-based method. The final dataset used in this analysis included 1691 individuals in 164 separated pedigrees and their summary statistics are provided in Table 3. On average, about 10% of offspring have missing genotypes and 10–20% have missing phenotypes of interest. As often is the case, the missing rates of genotypes in founders are higher, ranging from 10.5% to 23.5% for different sized families and the majority of the founders do not have phenotypes.

**Table 3:** Summary of GOLDN Family Data.

Family Size	# of families	Mean # of founders (Males/Females)	Mean # of offspring (Male/Female)	Mean # of being genotyped (founder/offspring)	Mean # of members having phenotypes		
					glucose	insulin	HOMA
3–6	54	2.30(1.17/1.13)	2.39(1.04/1.35)	2.78(0.54/2.24)	2.31	2.31	2.26
7–9	32	3.47(1.69/1.78)	4.69(2.22/2.47)	4.97(0.72/4.25)	4.25	4.22	4.19
10–12	30	4.43(2.23/2.20)	6.70(3.40/3.30)	6.33(0.60/5.73)	4.93	4.93	4.93
13–16	21	5.48(3.24/2.24)	8.86(4.24/4.62)	9.19(1.14/8.05)	7.81	7.81	7.81
17–20	19	6.47(3.47/3.00)	12.53(6.47/6.05)	12.32(0.95/11.37)	10.32	10.21	10.11
20+	8	7.62(4.00/3.62)	15.00(7.50/7.50)	14.50(1.25/13.25)	13.12	13.12	13.00

There are three haplotype blocks (Figure S2) on chromosome 4, coinciding with the candidate genes. The MAF range from 0.069 to 0.416 and there is no significant departure from HWE based on the χ^2^ test.

Analyses of HOMA and insulin were adjusted for age, gender, BMI, center, and physical activities and analysis of glucose was adjusted for age, gender, BMI, and smoking status (current smoker or not). The estimated parent-of-origin effect (*γ_2_*) for the three phenotypes and their p-values obtained from both models are listed in Table 4. We have found a strong parent-of-origin effect for glucose at the three loci of the microsomal triglyceride transfer protein (*MTP*) gene using the haplotype-based method, while the single-locus method is not powerful enough to raise the signal. Our findings complement previous studies. *MTP* is located in the lumen of the endoplasmic reticulum and is strictly necessary for the assembly and secretion of apolipoprotein B-containing lipoproteins [41], [42], [43]. Cell culture studies have suggested that *MTP* expression is positively regulated by glucose in primary hepatocytes [44] and negatively regulated by insulin and glucose in HepG2 cells [45].

**Table 4:** Parent-of-origin effect estimates (p-values) in genes *FABP2* and *MTP* on chromosome 4.

TestingSNP (MAF)	Glucose		HOMA		Insulin	
	Using a locus	Using haplotype	Using a locus	Using haplotype	Using a locus	Using haplotype
MTP_M1498 (0.338)	1.242 (0.4801)	1.039 (0.0043*)	0.090 (0.6957)	0.083 (0.0749)	−0.640 (0.4024)	−0.600 (0.0144)
MTP_M493 (0.214)	1.529 (0.4364)	1.490 (0.0039*)	−0.204 (0.4218)	0.014 (0.0584)	−1.028 (0.2136)	−0.953 (0.0091)
MTP_CYS174CYS (0.064)	−0.259 (0.9362)	−0.655 (0.0051*)	−0.148 (0.7101)	0.421 (0.0399*)	−0.745 (0.5860)	−1.737 (0.0081)
FABP2_A55S (0.234)	0.535 (0.7925)	0.242 (0.9061)	0.214 (0.3474)	0.230 (0.3114)	−0.983 (0.1990)	−0.989 (0.1912)
FABP2_M193 (0.417)	0.611 (0.7036)	0.749 (0.6440)	0.068 (0.7155)	0.130 (0.4815)	−0.532 (0.3943)	−0.769 (0.2144)
FABP2_M767 (0.234)	0.535 (0.7925)	0.242 (0.9061)	0.214 (0.3474)	0.230 (0.3114)	−0.983 (0.1990)	−0.989 (0.1912)

For this candidate gene based exploratory study, the p-values are significant even adjusting for a total of 11 tests. Please note that for the genome-wise assessment, the significance threshold can be much smaller after adjusting for the total number of SNP loci being tested.

Table 5 gives the estimates of haplotype frequencies using our haplotype-based method and HaploView. Our estimates from the three distinctive models are consistent up to the 2^nd^ decimal point, but slightly differ from the estimates by HaploView. Considering the estimates given by HaploView are based on founders only, we believe our estimates should be more accurate by incorporating all individuals' genotype information.

**Table 5:** Haplotype frequency estimates from Haploview and our method.

Haplotypes	Haploview	Model for Glucose	Model for HOMA	Model for Insulin
1^st^ Haplotype Block (MTP_M493, MTP_CYS174CYS, MTP_M1498)				
121	0.677	0.662	0.661	0.661
311	0.203	0.186	0.185	0.185
321	0.044	0.089	0.089	0.089
313	0.042	0.028	0.028	0.028
323	0.033	0.036	0.036	0.036
2^nd^ Haplotype Block (FABP2_A55S, FABP2_M193, FABP2_M767)				
232	0.600	0.683	0.683	0.683
414	0.209	0.234	0.234	0.234
212	0.191	0.083	0.083	0.083

## Discussion

In this paper, we have developed a method for testing parent-of-origin effect that can incorporate haplotype information to infer the parents of origin of testing alleles. Our method can accommodate large pedigree data, adjust for covariates, and allow for missing genotypes and phenotypes. We have demonstrated apparent power gains compared with the traditional single-locus approach in many realistic scenarios and our estimates are consistent and unbiased across all the experiments.

Our simulation studies also demonstrated that the method using haplotypes is more capable of recovering missing data than the method using the testing locus only. For modest random missing around 10%, the haplotype-based method can almost recover all the power if there are three or more siblings.

Most of our simulations were based on nuclear families. To evaluate the performance of our method in large pedigrees, we also conducted additional simulation based on the real families in the GOLDN study and the simulation results are consistent with what we have found based on nuclear families. The power was greatly improved for large families compared with nuclear families, using either single locus or haplotype method. The gain using the haplotype-based method over the single-locus method was more substantial at smaller heritability (h = 5%) than at larger heritability (h = 10%), indicating the haplotype method might be much more preferable than the single locus method in real studies, where heritability with respect to a single SNP/gene is usually low.

Exploring different study designs for optimal power to detect parent-of-origin given a fixed number of individuals, we have found that the best strategy is to recruit one parent and as many offspring as possible.

A more complicated model can have an additional term β_d_ I(g_p_≠g_m_) where the dominant effect β_d_ is reflected by the departure of mean phenotype in those with heterozygous genotypes. But the dominant effect can confound the parent-of-origin effect because the heterozygous group contains those individuals with ambiguous parents of origin. Instead of using a saturated model, we would suggest others investigate these two effects separately.

Although we have developed the model for quantitative traits, our method can be easily extended for binary and ordinal traits within the framework of generalized linear models. Our model can be easily incorporated into other software such as Merlin, which already has implemented the Elston-Stewart method to infer haplotypes.

Our model assumes random mating and HWE for haplotypes. One should check the validity of the assumptions before applying the method. Under the serious violation of the assumptions, the parameter may be biased and type I error rates inflated. In case HWE is violated, the diplotype frequencies instead of the haplotype frequencies can be used in the likelihood to relax the assumption.

We consider all compatible haplotype configurations with minimum recombinants or without recombinants. This is only applicable for tightly linked markers. For a set of sparsely spaced markers, a haplotype configuration with recombinants is more likely to occur, and in that case our method would need to be revised to accommodate the recombination probability.

Our method is computationally intensive. The computational complexity increases linearly with the size of pedigrees and linearly with the number of haplotypes. Therefore, the method is good to handle large pedigrees with a moderate number of haplotypes within a selected block. The number of SNPs needed to reach optimal power gain is region specific and data specific, as it depends on the haplotype structure of the surrounding SNPs and the pedigree structure. According to our simulations, in moderate families, a haplotype block with 2–3 SNPs that have moderate R^2^ (between 0.3–0.7) with the testing SNP might be sufficient to be utilized to improve the inference of the parent-of-origin of the testing alleles and thus the power. The power gain is minimal when the length of haplotype block increases more. For large-scale GWAS, we would suggest a sliding window method with short haplotype blocks to save the time in searching and determining the haplotype blocks.

## Supporting Information

Figure S1 **Power of detecting parent-of-origin effect with and without missing genotypes at loci 1–4.** Three scenarios are considered: no missing, (i) missing one parent's genotype in each family, (ii) for 10% random genotype missing, shown in blue, red, and green, respectively. Results connected by solid lines are obtained by the method using haplotypes, and the results connected by dotted lines are obtained by the method using the testing locus only.
(EPS)

Figure S2 **Haplotype structure of typed SNPs on chromosome 4. The numerical values within each square are R^2^.**
(EPS)

Table S1 **Type I errors of test of parent-of-origin in nuclear families with different family sizes, using different haplotype block length, and different missing mechanisms at α = 0.01.**
(DOC)

Table S2 **Haplotype frequencies, R^2^, and minor allele frequencies of the SNPs in GPX1.**
(DOC)

Appendix S1 **The Expectation for the Variance Components in the EM Algorithm.**
(DOC)

Appendix S2 **Revised Elston-Stewart Algorithm.**
(DOC)
