# Correction: A Powerful Test of Parent-of-Origin Effects for Quantitative Traits Using Haplotypes

**DOI:** 10.1371/annotation/3d15d176-7930-47b0-ba24-0a69d12fbeee

**Published:** 2012-07-09

**Authors:** Rui Feng, Yinghua Wu, Gun Ho Jang, Jose M. Ordovas, Donna Arnett

The grant information from the National Institutes of Health should read: This work is supported by National Institutes of Health grant R01GM088566 and R01ES016626.

In the original publication, a critical reference regarding the methods and simulation setup employed in our research was omitted. The additional reference is:

Lin D, Feng R, Chen J (2010) Maximum Likelihood Methods for Assessing Genetic Imprinting Using Case-Control Mother-Child Pair Data. Manuscript.

This work should be cited in the following Sections:

-Section 3 (Likelihood), page 3, 1st column, line 2 from the bottom, cite this reference and it should read as “…the parent of origin of the allele at the testing locus (Lin et al., 2010).”

-Section 7 (Simulations) first paragraph, page 4, 2nd column, cite Lin et al. (2010) at the end of the 2rd sentence. It should read as “…on the power of the haplotype-based method (Lin et al., 2010).”

-Section 7 (Simulations), page 4, 3rd paragraph, 1st sentence, add “following a similar simulation setup as in Lin et al. (2010)” before “we generated the genotypes…”

-Page 8, 1st column, line 8, add “A similar power gain by incorporating the haplotype information was also observed in Lin et al. (2010) in case-control mother-child pairs.” 

