# Supplementary material for: A Powerful Test of Parent-of-Origin Effects for Quantitative Traits Using Haplotypes
Source: PLoS One. 2011 Dec 13;6(12):e28909. doi: 10.1371/journal.pone.0028909 (PMC3236760; doi:10.1371/journal.pone.0028909)
Supplement: Table S1 — Type I errors of test of parent-of-origin in nuclear families with different family sizes, using different haplotype block length, and different missing mechanisms at α = 0.01. (DOC) [file pone.0028909.s003.doc]

Table S1. Type I errors of test of parent-of-origin in nuclear families with different family sizes, using different haplotype block length, and different missing mechanisms at α=0.01.

| Family size | Missing Mechanism | Single- locus Method | Haplotype-based method using different numbers of markers, L | | |
| --- | --- | --- | --- | --- | --- |
|  |  |  | L=2 | L=3 | L=4/5 |
| 1 offspring | (i) no missing | 0.011 | 0.010 | 0.009 | 0.009 |
|  | (ii) 10% random missing | 0.009 | -- | -- | 0.011 |
|  | (iii) one parent missing | 0.009 | -- | -- | 0.009 |
| 2 offspring | (i) no missing | 0.010 | 0.010 | 0.011 | 0.009 |
|  | (ii) 10% random missing | 0.011 | -- | -- | 0.010 |
|  | (iii) one parent missing | 0.011 | -- | -- | 0.010 |
| 3 offspring | (i) no missing | 0.011 | 0.010 | 0.010 | 0.008 |
|  | (ii) 10% random missing | 0.010 | -- | -- | 0.011 |
|  | (iii) one parent missing | 0.010 | -- | -- | 0.011 |
| 4 offspring | (i) no missing | 0.010 | 0.010 | 0.010 | 0.010 |
|  | (ii) 10% random missing | 0.008 | -- | -- | 0.008 |
|  | (iii) one parent missing | 0.008 | -- | -- | 0.009 |
