# Supplementary material for: A Powerful Test of Parent-of-Origin Effects for Quantitative Traits Using Haplotypes
Source: PLoS One. 2011 Dec 13;6(12):e28909. doi: 10.1371/journal.pone.0028909 (PMC3236760; doi:10.1371/journal.pone.0028909)
Supplement: Table S2 — Haplotype frequencies, R2, and minor allele frequencies of the SNPs in GPX1. (DOC) [file pone.0028909.s004.doc]

Table S2. Haplotype frequencies, R^2^, and minor allele frequencies of the SNPs in GPX1.

| Haplotype | Frequency | R^2^ | | | | | | MAF |
| --- | --- | --- | --- | --- | --- | --- | --- | --- |
| 00001 | 0.298 | SNP | 1 | 2 | 3 | 4 | 5 |  |
| 00000 | 0.267 | 1 | 1 | 0.781 | 0.013 | 0.794 | 0.184 | 0.183 |
| 00101 | 0.152 | 2 |  | 1 | 0.004 | 0.549 | 0.144 | 0.149 |
| 11010 | 0.117 | 3 |  |  | 1 | 0.070 | 0.012 | 0.283 |
| 00100 | 0.099 | 4 |  |  |  | 1 | 0.146 | 0.151 |
| 10010 | 0.034 | 5 |  |  |  |  | 1 | 0.450 |
| 11100 | 0.032 |  |  |  |  |  |  |  |
