# Supplementary material for: A Powerful Test of Parent-of-Origin Effects for Quantitative Traits Using Haplotypes
Source: PLoS One. 2011 Dec 13;6(12):e28909. doi: 10.1371/journal.pone.0028909 (PMC3236760; doi:10.1371/journal.pone.0028909)
Supplement: Appendix S1 — The Expectation for the Variance Components in the EM Algorithm. (DOC) [file pone.0028909.s005.doc]

**Appendix S1**

**The Expectation for the Variance Components in the EM Algorithm**

In model (2) $Y_{j}^{i}=\mathbf{x}_{j}^{i}\mathbf{\beta}+(g_{p,j}^{i}+g_{m,j}^{i})\gamma_{1}+(g_{p,j}^{i}-g_{m,j}^{i})\gamma_{2}+U_{r}^{i}+\varepsilon_{j}^{i},\text{ for }i=1,...,n\text{, }j=1,...,n_{i}.$

The posterior likelihood, given $U_{r}^{i}$ and current parameter estimates $\mathbf{\beta}_{k}$, $\gamma_{1,k}$, $\gamma_{2,k}$ and $\rho_{k}$, is

where $N=\sum_{i=1} n_{i}$, $\tilde{g}^{i}=\sum_{(h_{p}^{i},h_{m}^{i})\sim G_{f}^{i}} \left[(g_{p}^{i}+g_{m}^{i})\gamma_{1,k}-(g_{p}^{i}-g_{m}^{i})\gamma_{2,k}\right]p\left((h_{p}^{i},h_{m}^{i})|G_{f}^{i}\right)$

Denote$\theta=(\mathbf{\beta},\gamma,\mathbf{\rho},\sigma_{1},\sigma_{2})$ Then the expectation of $l(Y|U)$ with respect to $p(U|Y,\hat{\theta}_{k})$is the augmented expectation, given by

(A.1)

Replacing (a.2)-a(.4) back to (A.1), $Q(\sigma_{1},\sigma_{2}|\hat{\theta}_{k})$ becomes

$$\begin{matrix} Q(\sigma_{1},\sigma_{2}|\hat{\theta}_{k}) \\ =-\frac{N}{2}\log\left(2\pi\sigma_{2}^{2}\right)-\frac{1}{2\sigma_{2}^{2}}\sum_{i} \left(Y^{i}-\mathbf{x}^{i}\hat{\mathbf{\beta}}_{k}-\tilde{g}^{i}\right)^{'}\left(Y^{i}-\mathbf{x}^{i}\hat{\mathbf{\beta}}_{k}-\tilde{g}^{i}\right)+\frac{1}{\sigma_{2}^{2}}\sum_{i} \left(Y^{i}-\mathbf{x}^{i}\hat{\mathbf{\beta}}_{k}-\tilde{g}^{i}\right)^{'}E_{U|Y,\hat{\theta}}(U_{r}^{i}) \\ -\frac{1}{2\sigma_{2}^{2}}\sum_{i} \left[E_{U|Y,\hat{\theta}}(U_{r}^{i})\right]^{'}E_{U|Y,\hat{\theta}}(U_{r}^{i})-\frac{1}{2}\sum_{i} \log\left|2\pi\sigma_{1}^{2}\phi^{i}\right|-\frac{1}{2\sigma_{1}^{2}}\left[E_{U|Y,\hat{\theta}}(U_{r}^{i})\right]^{'}(\phi^{i})^{-1}E_{U|Y,\hat{\theta}}(U_{r}^{i}) \\ -\frac{1}{2}trace\left[\left(\frac{1}{\sigma_{2}^{2}}I_{n_{i}}+\frac{1}{\sigma_{1}^{2}}(\phi^{i})^{-1}\right)\left(\frac{1}{\hat{\sigma}_{2,(k-1)}^{2}}I_{n_{i}}+\frac{1}{\hat{\sigma}_{1,(k-1)}^{2}}(\phi^{i})^{-1}\right)^{-1}\right]\text{ (A.5)} \end{matrix}$$

Denote the singular value decomposition of the kinship matrix $\phi^{i}$as where $D^{i}$ is a diagonal matrix with the diagonal elements $d_{1}^{i},....,d_{n_{i}}^{i}$. Then the last part of (A.5) becomes

$$\begin{matrix} \frac{1}{2}trace\left[\left(\frac{1}{\sigma_{2}^{2}}I_{n_{i}}+\frac{1}{\sigma_{1}^{2}}(\phi^{i})^{-1}\right)\left(\frac{1}{\hat{\sigma}_{2,(k-1)}^{2}}I_{n_{i}}+\frac{1}{\hat{\sigma}_{1,(k-1)}^{2}}(\phi^{i})^{-1}\right)^{-1}\right] \\ =\frac{1}{2}trace\left[\left(\frac{1}{\sigma_{2}^{2}}I_{n_{i}}+\frac{1}{\sigma_{1}^{2}}(D^{i})^{-1}\right)\left(\frac{1}{\hat{\sigma}_{2,(k-1)}^{2}}I_{n_{i}}+\frac{1}{\hat{\sigma}_{1,(k-1)}^{2}}(D^{i})^{-1}\right)^{-1}\right] \\ =\frac{1}{2}\sum_{j}^{n_{i}} \frac{\frac{1}{\sigma_{2}^{2}}+\frac{1}{\sigma_{1}^{2}d_{j}^{i}}}{\frac{1}{\hat{\sigma}_{2,(k)}^{2}}+\frac{1}{\hat{\sigma}_{1,(k)}^{2}d_{j}^{i}}} \end{matrix}$$

Replace this in Q and take the first derivative of Q with respect to $\sigma_{1}$ and $\sigma_{2}$, we will have

$$\begin{matrix} \frac{\partial Q(\sigma_{1},\sigma_{2}|\hat{\theta}_{k})}{\partial\sigma_{2}} \\ =-\frac{N}{2\sigma_{2}}+\frac{1}{\sigma_{2}^{3}}\sum_{i} \left(Y^{i}-\mathbf{x}^{i}\hat{\mathbf{\beta}}_{k}-\tilde{g}^{i}-E_{U|Y,\hat{\theta}}(U_{r}^{i})\right)^{'}\left(Y^{i}-\mathbf{x}^{i}\hat{\mathbf{\beta}}_{k}-\tilde{g}^{i}-E_{U|Y,\hat{\theta}}(U_{r}^{i})\right)+\sum_{i} \frac{1}{\left(\frac{1}{\hat{\sigma}_{2,(k)}^{2}}+\frac{1}{\hat{\sigma}_{1,(k)}^{2}d_{i}}\right)\sigma_{2}^{3}}\text{ } \\ \text{=0 } \end{matrix}$$

Solve for $\sigma_{2}$, we have

$$\begin{matrix} \hat{\sigma}_{2,(k)}^{2}=\frac{1}{N}\sum_{i} \left(Y^{i}-\mathbf{x}^{i}\hat{\mathbf{\beta}}_{k}-\tilde{g}^{i}-E_{U|Y,\hat{\theta}}(U_{r}^{i})\right)^{'}\left(Y^{i}-\mathbf{x}^{i}\hat{\mathbf{\beta}}_{k}-\tilde{g}^{i}-E_{U|Y,\hat{\theta}}(U_{r}^{i})\right)+\frac{1}{N}\sum_{i} \sum_{j}^{n_{i}} \frac{1}{\frac{1}{\hat{\sigma}_{2,(k)}^{2}}+\frac{1}{\hat{\sigma}_{1,(k)}^{2}d_{j}^{i}}}\text{ } \\ \text{=}\frac{1}{N}\left(\sum_{i} \left(Y^{i}-\mathbf{x}^{i}\mathbf{\beta}_{k}-\tilde{g}-\tilde{u}^{i}\right)^{'}\left(Y^{i}-\mathbf{x}^{i}\mathbf{\beta}_{k}-\tilde{g}-\tilde{u}^{i}\right)+\sum_{i} \sum_{j}^{n_{i}} \frac{1}{\frac{1}{\hat{\sigma}_{2,(k-1)}^{2}}+\frac{1}{d_{j}^{i}\hat{\sigma}_{1,(k-1)}^{2}}}\right) \end{matrix}$$

Similarly, we can have

$$\hat{\sigma}_{1,(k)}^{2}=\frac{1}{N}\left(\sum_{i} \tilde{u}'_{i}(\phi^{i})^{-1}\tilde{u}_{i}+\sum_{i} \sum_{j}^{n_{i}} \frac{1}{\frac{d_{j}^{i}}{\hat{\sigma}_{2,(k-1)}^{2}}+\frac{1}{\hat{\sigma}_{1,(k-1)}^{2}}}\right)$$

where $\tilde{g}^{i}=\sum_{(h_{p}^{i},h_{m}^{i})\sim G_{f}^{i}} \left[(g_{p}^{i}+g_{m}^{i})\gamma_{1,k}-(g_{p}^{i}-g_{m}^{i})\gamma_{2,k}\right]p\left((h_{p}^{i},h_{m}^{i})|G_{f}^{i}\right)$ and $\tilde{u}^{i}=\left(\frac{1}{\tilde{\sigma}_{2,(k-1)}^{2}}I_{n{}_{i}}+\frac{1}{\tilde{\sigma}_{1,(k-1)}^{2}}(\phi^{i})^{-1}\right)^{-1}\frac{1}{\tilde{\sigma}_{2,(k-1)}^{2}}Y_{i}$. #
