# Supplementary material for: A Powerful Test of Parent-of-Origin Effects for Quantitative Traits Using Haplotypes
Source: PLoS One. 2011 Dec 13;6(12):e28909. doi: 10.1371/journal.pone.0028909 (PMC3236760; doi:10.1371/journal.pone.0028909)
Supplement: Appendix S2 — Revised Elston-Stewart Algorithm. (DOC) [file pone.0028909.s006.doc]

**Appendix S2**

Revised Elston-Stewart Algorithm

1. Starting from all individuals in the last generation, for individual *k* let $J_{k}(H_{k})=1$.

2. Then for the previous generation, calculate the conditional probability for a particular combination of two haplotypes, *H_k_*, of individual *k*, his/her spouses and their offspring., i.e.,

(A.1)

where *G_j_* denotes the genotype of individual *j*, $P(Y_{o}|H_{o})$ is the probability that can be determined by our model (2) and $P(H_{o}|H_{j},H_{k})$ is the transmission probability.

These two probabilities are the key differences between Eq. (A.1) and the traditional E-S method originally used in linkage analysis. In our revised implementation, the “penetrance” probability can be expanded:$\begin{matrix} P(Y_{o}|H_{o})=P(Y_{o}|(h_{o},h'_{o}))=P(Y_{o}|(g_{po}^{t},g_{mo}^{t})\cup G_{po}^{\backslash t},G_{mo}^{\backslash t}) \\ =\frac{P\left(G_{po}^{\backslash t},G_{mo}^{\backslash t}|Y_{0}\cup g_{po}^{t},g_{mo}^{t}\right)P(Y_{o}|(g_{po}^{t},g_{mo}^{t})}{P\left(G_{po}^{\backslash t},G_{mo}^{\backslash t}|g_{po}^{t},g_{mo}^{t}\right)} \\ =P\left(Y_{o}|(g_{po},g_{mo})\right)\left|_{(g_{po},g_{mo})\in(h_{o},h'_{o})}\right. \end{matrix}$

where$(g_{po}^{t},g_{mo}^{t})$ are the sourced genotypes at the testing locus of this offspring and $G_{po}^{\backslash t},G_{mo}^{\backslash t}$ are the sourced genotype combination of non-testing loci, $h_{o}$is composed by $g_{po}^{t}$ and $G_{po}^{\backslash t}$, and $h'_{o}$is composed by $g_{mo}^{t}$ and $G_{mo}^{\backslash t}$. The last line in the equation results from $G_{po}^{\backslash t},G_{mo}^{\backslash t}$ being independent of Y at none testing loci. Thus the probability is determined by both haplotypes and their origins and is a function of all covariate effects, γ_1_ and γ_2_. In association studies, γ_2_ is irrelevant, so, the sources of haplotypes do not matter.

The transmission probability $P(H_{o}|H_{j},H_{k})$in Eq. (A.1) is the conditional probability of sourced haplotype given parental non-sourced haplotypes; while in the traditional method, it is the conditional probability of genotypes given parental genotypes without considering the parents of origin of the alleles.

3. Repeat step 2 until all founders and their possible haplotypes have been exhausted.

4. Take the sum of all likelihood for all families, and maximize it with respect to parameters β and *ρ_1_,….., ρ_t_*.
